# Supplementary material for: The interactome of intact mitochondria by cross-linking mass spectrometry provides evidence for coexisting respiratory supercomplexes
Source: Mol Cell Proteomics. 2017 Dec 8;17(2):216–32. doi: 10.1074/mcp.RA117.000470 (PMC5795388; doi:10.1074/mcp.RA117.000470)
Supplement: Supplemental Data [file supp_RA117.000470_133922_0_supp_23978_fzffwf.zip › spectra_annotation/mito_spectra_annotation/483-1-1-1-4-1.pdf]

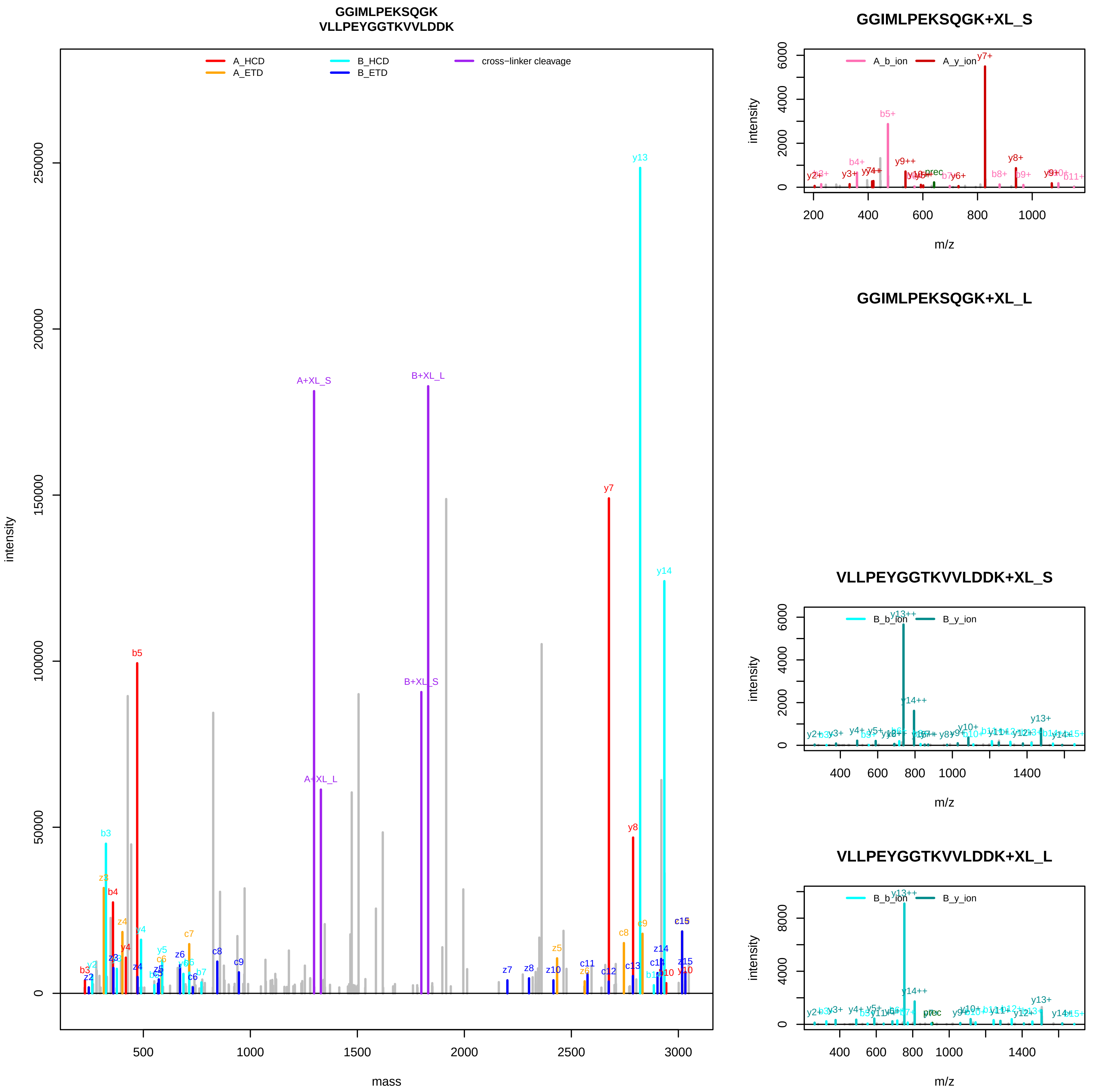
GGIMLPEKSQGK
VLLPEYGGTKVVLDDK
A_HCD
A_ETD
B_HCD
B_ETD
cross−linker cleavage
A+XL_S
B+XL_L
B+XL_S
A+XL_L
y13
y14
y7
y8
b5
b4
b3
y4
z4
z3
c7
c8
c9
z5
z7
z8
z10
c11
c12
c13
c14
z14
c15
z15
y10
intensity
mass
0
50000
100000
150000
200000
250000
500
1000
1500
2000
2500
3000
GGIMLPEKSQGK+XL_S
A_b_ion
A_y_ion
y7+
b5+
y8+
y9++
b4+
intensity
m/z
GGIMLPEKSQGK+XL_L
VLLPEYGGTKVVLDDK+XL_S
B_b_ion
B_y_ion
y13++
y14++
y13+
VLLPEYGGTKVVLDDK+XL_L
0
2000
4000
6000
8000
200
400
600
800
1000
1400
